# Correlation of examiner judgement and radiological digital pictures in infants with upper cervical spine dysfunction: a cross-sectional study

**DOI:** 10.1007/s00247-026-06718-8

**Published:** 2026-07-11

**Authors:** Jan Peter, Miklos Csato, Inga Paravicini, Martina Wehrli, Petra Schweinhardt, Anke Langenfeld

**Affiliations:** 1 Department of Chiropractic Medicine, Balgrist University Hospital and University of Zurich, Zurich, Switzerland; 2 Department of Radiology, Balgrist University Hospital and University of Zurich, Zurich, Switzerland

**Keywords:** Imaging, Infants, Open mouth odontoid view, Upper cervical spine dysfunction

## Abstract

**Background:**

This study aimed to (1) assess the inter-rater reliability of lateral atlantodental distance measurements in digital open-mouth radiographs of infants with upper cervical spine dysfunction, and (2) investigate correlations between lateral atlantodental distance values and clinical findings.

**Methods:**

72 digital radiographs of the odontoid region from infants aged 3–12 months were analyzed (Aug 2021–Dec 2023). Lateral atlantodental distance was measured bilaterally by three independent raters. Inter-rater reliability and measurement precision was assessed using intraclass correlation coefficients (ICC), standard error of measurement (SEM), and minimal detectable change (MDC). Side differences were evaluated using paired t-tests. Mean lateral atlantodental distance values were further examined for their association with the clinically assessed side of cervical rotational malposition using logistic regression and receiver operating characteristic (ROC) analysis.

**Results:**

Inter-rater reliability was excellent for right (ICC=0.91 [95% CI: 0.86 – 0.94]) and left (ICC=0.91 [95% CI: 0.86 – 0.95]). The SEM was 0.62 mm for both sides, the MDC was 1.72 mm (right) and 1.73 mm (left). In infants with left-sided cervical rotational malposition, lateral atlantodental distance was significantly greater on the left side, whereas in infants with right-sided cervical rotational malposition, lateral atlantodental distance was significantly greater on the right side. Right-sided lateral atlantodental distance was a significant predictor of clinically assessed right-sided cervical rotational malposition (OR 1.80, 95% CI 1.14–2.84, *P*=0.01). ROC analysis demonstrated moderate discriminatory performance (AUC = 0.74), meaning that the model is reasonably capable of distinguishing patients with right-sided cervical rotational malposition from those without.

**Conclusions:**

Although lateral atlantodental distance measurements showed excellent inter-rater reliability, the SEM was relatively large compared with the small absolute values of the measurements (in millimeters), which limits the ability to detect subtle side-to-side asymmetries. Lateral atlantodental distance should be interpreted as a reproducible radiographic finding associated with cervical rotational malposition rather than as an independent diagnostic marker or pathognomonic sign in infants.

**Trial registration:**

ClinicalTrials.gov ID NCT04981782 (28.07.2021).

**Graphical Abstract:**

**Figure:**
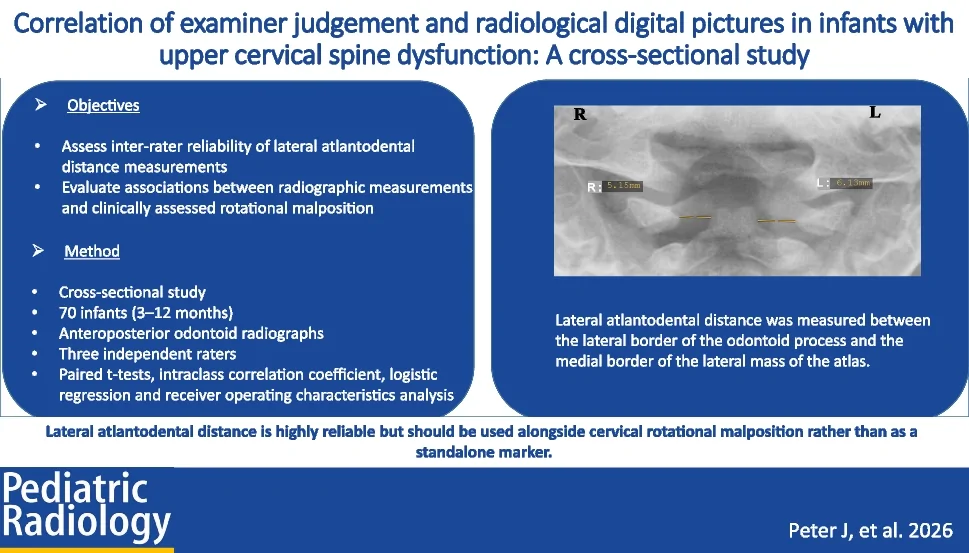

**Supplementary Information:**

The online version contains supplementary material available at https://doi.org/10.1007/s00247-026-06718-8.

## Background

Torticollis, a condition defined by a persistent head tilt and rotated neck posture, affects up to 16% of newborns and can be broadly classified into muscular and non-muscular types [1]. The most common presentation is congenital muscular torticollis, typically resulting from fibrosis or contracture of the sternocleidomastoid muscle [2]. Perinatal factors such as in-utero positioning, vacuum-assisted delivery, or prolonged labor are considered relevant risk factors. These mechanical influences can also contribute to cranial asymmetry and scoliosis, whereas positional plagiocephaly is generally regarded as a secondary consequence of torticollis [1, 3–5]. Non-muscular torticollis, while less frequently diagnosed, comprises a range of underlying causes including neurological conditions, infections, tumors, or dysfunctions of the auditory or vestibular systems [6–9]. Additionally, underlying causes in the function of the upper cervical spine are discussed in literature [4, 5]. Furthermore, different theoretical frameworks such as Kinematic Imbalance due to Suboccipital Strain syndrome [10, 11], arthrogenic torticollis [5], and upper cervical spine dysfunction [12, 13] are used in daily clinical practice to explain symptoms related to torticollis such as postural asymmetry, and restricted cervical range of motion in the absence of muscular contracture in infants. Overlapping clinical pictures within these theoretical frameworks reflect ongoing uncertainty regarding the underlying mechanisms of torticollis and pose challenges for both research and clinical decision-making. To date, no universally accepted definition or diagnostic protocol exists for non-muscular forms of torticollis, and the clinical relevance of proposed dysfunctions in the upper cervical region remains debated [11, 14]. Clarifying these conceptual distinctions and their empirical basis is essential for improving the consistency of future studies and for avoiding premature assumptions about causality or clinical significance.

Imaging is generally used sparingly in the diagnostic workup of torticollis in infants. Boyko et al. (2017) reported that imaging was rarely performed as part of routine evaluation [15]. While magnetic resonance imaging may be considered in selected cases to rule out neurological abnormalities, it is not typically part of standard protocols due to cost, limited availability, and the need for sedation in young infants [15]. The odontoid view (also known as the open-mouth view) is a specific radiographic technique used to visualize the odontoid process (dens axis) and the atlantoaxial joint in the upper cervical spine. It is valuable for detecting fractures, deformities, or instabilities and other bony abnormalities of the upper cervical spine with any suspected disease such as Down or Klippel Feil Syndrome [16, 17]. One parameter assessed using this view is the lateral atlantodental distance which refers to the space between the lateral edge of the dens (C2) and the inner margin of the atlas (C1) on either axial or coronal images. Asymmetry in this distance may indicate atlantoaxial instability, which can result from trauma, congenital malformation, or inflammatory conditions (e.g. rheumatoid arthritis or trisomy 21)[16, 17]. Obtaining a reliable odontoid view in infants is challenging due to small anatomical structures, limited cooperation, and the need to minimize radiation exposure [18, 19]. In addition, Driehuis et al. (2021) have pointed out that the interpretation of clinical examination findings in infants with suspected upper cervical spine dysfunction, such as range-of motion tests, remains subjective and insufficiently validated by robust clinical trials [13]. This highlights a diagnostic challenge in early infancy, with both imaging and clinical assessment facing limitations. In light of these diagnostic limitations, there is a need for objective, reproducible, and sensitive assessment tools to support clinical decision-making in suspected upper cervical spine dysfunction.

The aim of this cross-sectional study is to assess the inter-rater reliability (ICC) of the odontoid view in lateral lateral atlantodental distance measurements and to explore associations between lateral atlantodental distance measurements and clinical presentation of infants with suspected upper cervical dysfunction in order to support clinical decision-making in early infancy.

## Methods/study design

The study followed a cross-sectional design. Right and left lateral atlantodental distance measurements were obtained from radiographic images. Inter-rater agreement among three raters was analyzed, as well as the association between lateral atlantodental distance measurements and the clinician’s assessment of cervical rotational malposition. Ethical approval was obtained from the local ethics committee (BASEC Nr. 2020-02328). The primary study was registered at a public trial registry (NCT04981782). Data collection took place between August 2021 and December 2023.

This study is reported in accordance with the STROBE (Strengthening the Reporting of Observational Studies in Epidemiology) guidelines for cross-sectional studies. The completed STROBE checklist is included as Supplementary Material 1.

### Participants

Radiological digital pictures of the odontoid of infants were assessed which were included in another study (blinded for review) fulfilling the following inclusion criteria: diagnosis of upper cervical spine dysfunction, age between three and twelve months and signed consent of the legal representative. Excluded were infants with known illness other than torticollis including neurological impairments, previous neck trauma, previous neck surgery and developmental delay. Radiological imaging was performed as part of the clinical routine before mobilization of the upper cervical spine. This was done in order to rule out contraindications for chiropractic treatment such as malformations, instabilities or severe pathologies of the infants’ upper cervical spine. During radiographic imaging, infants were gently supported by a parent, who stabilized the infant’s trunk and pelvis, to minimize movement while avoiding any direct fixation of the neck or shoulders. An x-ray technician gently held the infants` head within the x-ray window without overcorrecting the infants` head position. Afterwards the infants were animated to open the mouth by the present parent and/or the x-ray technician.

All included infants were referred to chiropractic care by their pediatrician, with a presumptive diagnosis of an upper cervical spine dysfunction. Any other underlying diagnoses such as neurological causes were ruled out before referral to chiropractic care by the pediatrician. No additional diagnostic procedures were executed during chiropractic care.

For the present study, infant age, sex, birth weight, birth length, the clinically assessed predominant side of cervical rotational malposition, and side-specific lateral atlantodental distance measurements were included in the analysis. Additional demographic, perinatal, and parental observational variables were collected as part of the primary study and are reported elsewhere (blinded for review).

### Examination

During the physical evaluation, the treating clinician assessed the presence and predominant direction of cervical rotational malposition. For the primary side-specific analyses, infants were categorized according to the clinically determined predominant rotational malposition as either right-sided or left-sided.

### Rating

Radiograph adequacy and assessability were determined independently by each rater according to a predefined standard operating procedure (SOP) (Supplementary Material 2). Raters were blinded to each other’s assessments.

Rater one (18 years of clinical experience) and rater three (7 years of clinical experience) were clinicians specialized in musculoskeletal care. Rater two was a radiologist with 4 years of experience in musculoskeletal imaging. Before image assessment, all raters completed two standardized training sessions on upper cervical spine radiograph evaluation and lateral atlantodental distance measurement.

**Fig. 1:**
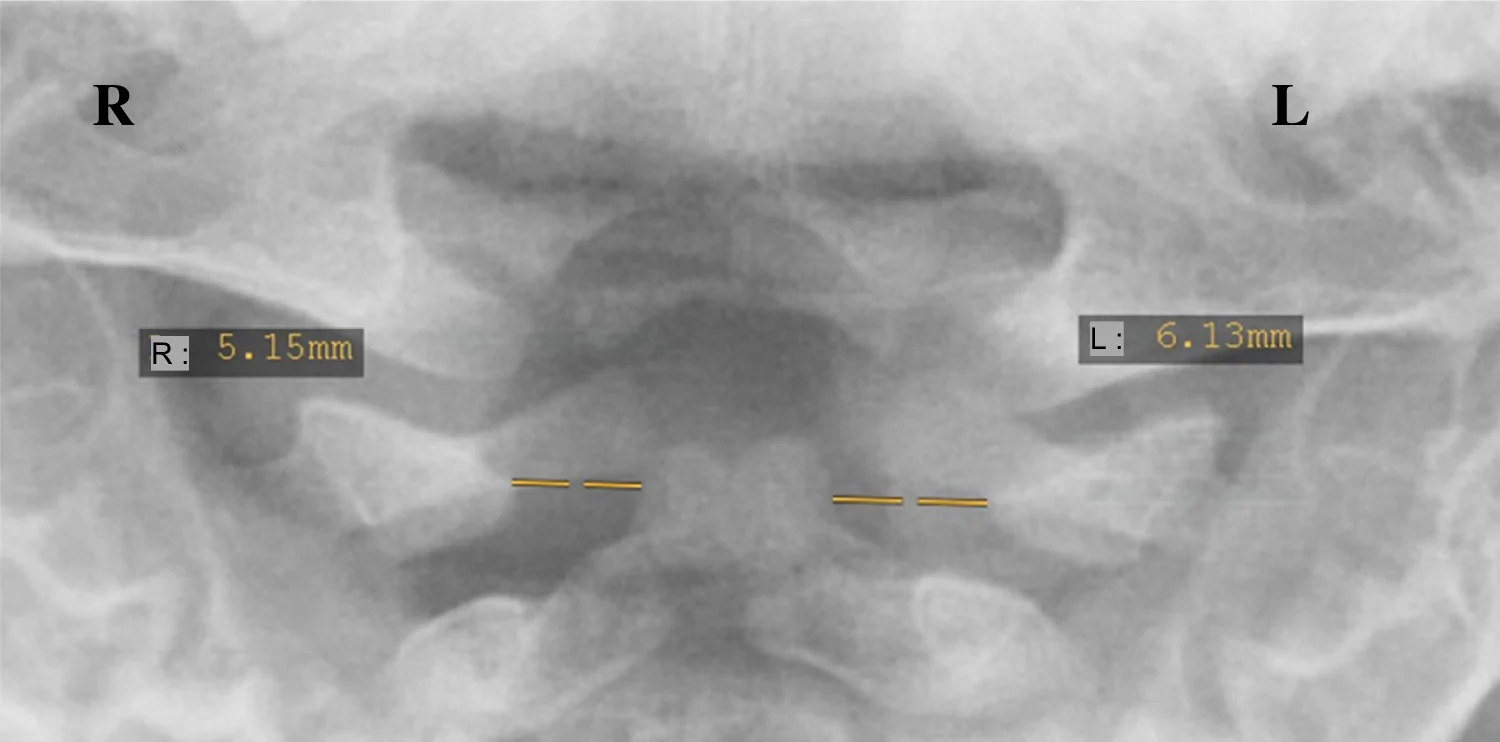
Measurement of lateral atlantodental distance on anteroposterior radiograph of an infant. The image is depicted in radiological convention – anatomical right appears on the left and anatomical left on the right. lateral atlantodental distance was measured between the lateral border of the odontoid process and the medial border of the lateral mass of the atlas (C1), using radiographic data obtained in this study and processed with digital software (MERLIN Diagnostic Workcenter, Phönix-PACS GmbH, Freiburg, Germany). Anatomical sides are indicated by the markers “R” and “L”

For image assessability, the outline of the odontoid process and the lateral masses of the atlas (C1) had to be clearly visible. The shortest distance between the lateral aspect of the dens (C2) and the medial border of the lateral mass of the atlas (C1) was measured on anterior-to-posterior radiographic views (Fig. 1) [20]. Measurements were recorded in millimeters for the right and left lateral atlantodental distance using the MERLIN Diagnostic Workcenter (Phönix-PACS GmbH, Freiburg, Germany).

### Data analysis

Study data was managed using REDCap Electronic Data Capture systems (Vanderbilt University, TN, USA) [21, 22]. Statistical analysis was performed using SPSS (Version 30, IBM, Armonk, NY USA). Demographics were determined and descriptive statistics of the sample were calculated. Paired-sampled t-tests were conducted to assess differences in lateral atlantodental distance between right and left sides [23]. In all t-tests, values in parentheses denote degrees of freedom. In addition, the data were subdivided based on the side of the infant’s cervical malposition (right- or left-sided), allowing side-specific comparisons. Inter-rater reliability (agreement between raters) was assessed using intraclass correlation coefficient (ICC) based on mean-rating (k=3), absolute-agreement, two-way mixed-effects model. The ICC was calculated separately for the measurements of the lateral atlantodental distance on the right and left sides. Values greater than 0.9 represent excellent reliability, 0.75—0.9 good agreement, 0.5—0.75 moderate agreement and under 0.5 poor agreement [24]. Furthermore, standard error of measurement (SEM) (SEM=SD √1 − ICC) was calculated to quantify the expected variability in individual scores due to measurement error [25]. Minimal detectable change (MDC) (MDD=1.96 ∗ √2 ∗ SE) were calculated to assess absolute reliability. Significance level was set at α=0.05 [26, 27]. In a separate analysis, the association between lateral atlantodental distance and cervical rotational malposition was examined. The data were stratified based on the clinically affected side. Specifically, left and right lateral atlantodental distance values were compared in infants with left-sided malposition and with right-sided malposition separately. This allowed for a side-specific analysis of asymmetry in relation to clinical findings.

For inferential analysis, logistic regression and receiver operating characteristic (ROC) analyses were performed to explore whether lateral atlantodental distance measurements were associated with the clinically assessed side of cervical rotational malposition [27, 28]. Right-sided cervical rotational malposition was specified as the outcome of interest in the logistic regression and ROC analyses. Mean lateral atlantodental distance values were calculated using all available measurements from the three raters. Cases with missing measurements from one or two raters were retained for analyses of mean left and right lateral atlantodental distance values using the available measurements. ICC analyses were restricted to complete cases with measurements available from all three raters.

To quantify side-specific asymmetry, the difference between the affected and unaffected side was calculated (AsymmetryOverall). Positive values indicated greater lateral atlantodental distance on the clinically affected side, whereas negative values indicated greater lateral atlantodental distance on the clinically unaffected side.

## Results

### Demographics and descriptive statistics

Seventy-two infants were initially included (mean age 145.4 (SD 29) days, mean birth weight 3303.6 (SD 542.3) g, mean birth length 49.3 (SD 2.8) cm). Forty-seven infants (65%) were male and twenty-five (35%) female. The clinician rated thirty-five (49%) infants as having a rotational malposition of the head to the right side and thirty-seven (51%) to the left side.

### Lateral atlantodental distance

Due to insufficient radiographic image quality, lateral atlantodental distance measurements could not be obtained in seven cases by rater 1, eleven cases by rater 2, and three cases by rater 3. Two radiographs were excluded entirely because image quality was insufficient for lateral atlantodental distance assessment by any rater. Both excluded cases had been clinically classified as left-sided cervical rotational malposition. Consequently, the final study sample for the side-specific lateral atlantodental distance analysis included 70 infants, comprising 35 with left-sided and 35 with right-sided cervical rotational malposition.

In infants with a left-sided rotational malposition (*n*=35), the left lateral atlantodental distance was significantly larger than the right. A paired-samples t-test revealed a significant side difference, t(34)=3.11, *P*=0.004, with a mean difference of 1.11 mm (95% CI: 0.39 to 1.84 mm) and a moderate effect size (Cohen’s d=0.53), supporting the presence of side-specific asymmetry within this subgroup.

In infants with a right-sided rotational malposition (*n*=35), the right lateral atlantodental distance was significantly greater than the left. A paired-samples t-test revealed a significant side difference, t(34) = −2.08, *P*=0.045, with a mean difference of −0.62 mm (95% CI: −1.22 to −0.01 mm) and a small to moderate effect size (Cohen’s d=0.35), indicating side-specific asymmetry within this subgroup.

Descriptive statistics for the lateral atlantodental distance measurements are presented in Table 1. The values illustrate the observed side-specific differences in lateral atlantodental distance according to the side of cervical rotational malposition. In infants with a left-sided malposition, the lateral atlantodental distance was greater on the left side, whereas in infants with a right-sided malposition, the lateral atlantodental distance was greater on the right side.

**Table 1:** Descriptive statistics for lateral atlantodental distance by side of cervical rotational malposition

Malposition side	Anatomical side	Mean (mm)	SD	*N*	95% CI
Left rotational malposition	Left	6.41	1.39	35	5.93–6.90
Left rotational malposition	Right	5.30	1.36	35	4.82–5.78
Right rotational malposition	Left	5.66	1.15	35	5.26–6.06
Right rotational malposition	Right	6.28	1.05	35	5.90–6.66

*CI*, confidence interval; *SD*, standard deviation.

“*Malposition Side*” refers to the side of cervical rotational malposition as assessed by the clinician;

“*Anatomical Side*” refers to the anatomical side (left or right) on which the was measured.

The asymmetry variable (AsymmetryOverall), calculated as the difference between the affected and unaffected side, showed both positive and negative values within both clinically defined subgroups.

### Interrater reliability, standard error of measurement and minimal detectable change

Based on 60 complete cases, interrater reliability (ICC) was excellent for both left-sided (0.91 [95% CI: 0.86–0.95]) and right-sided (0.91 [95% CI: 0.86–0.94]) lateral atlantodental distance measurements. For the left-side, SEM was 0.62 mm, and the minimal detectable change (MDC) was 1.73 mm. For the right-side, SEM was also 0.62 mm and MDC was 1.72 mm. These thresholds represent the minimum difference required to be confident that an observed change exceeds measurement error and reflects a true difference.

### Binary logistic regression

Binary logistic regression was performed in 70 infants with available side-specific mean lateral atlantodental distance measurements. The analysis demonstrated an association between side-specific lateral atlantodental distance measurements and clinically assessed cervical rotational malposition. Only the mean right-sided lateral atlantodental distance was a significant predictor of clinically assessed right sided malposition (OR 1.80, 95% CI 1.14–2.84, *P*=0.01), whereas the mean left-sided lateral atlantodental distance was not statistically significant (Table 2).

**Table 2:** Binary logistic regression model predicting right-sided cervical rotational malposition

Predictor	OR	95% CI	*P*-value
Mean left lateral atlantodental distance	0.70	0.45–1.09	0.12
Mean right lateral atlantodental distance	**1.80**	**1.14–2.84**	**0.01**

*OR*, odds ratio; *CI*, confidence interval. Significant results are highlighted in bold.

**Fig. 2:**
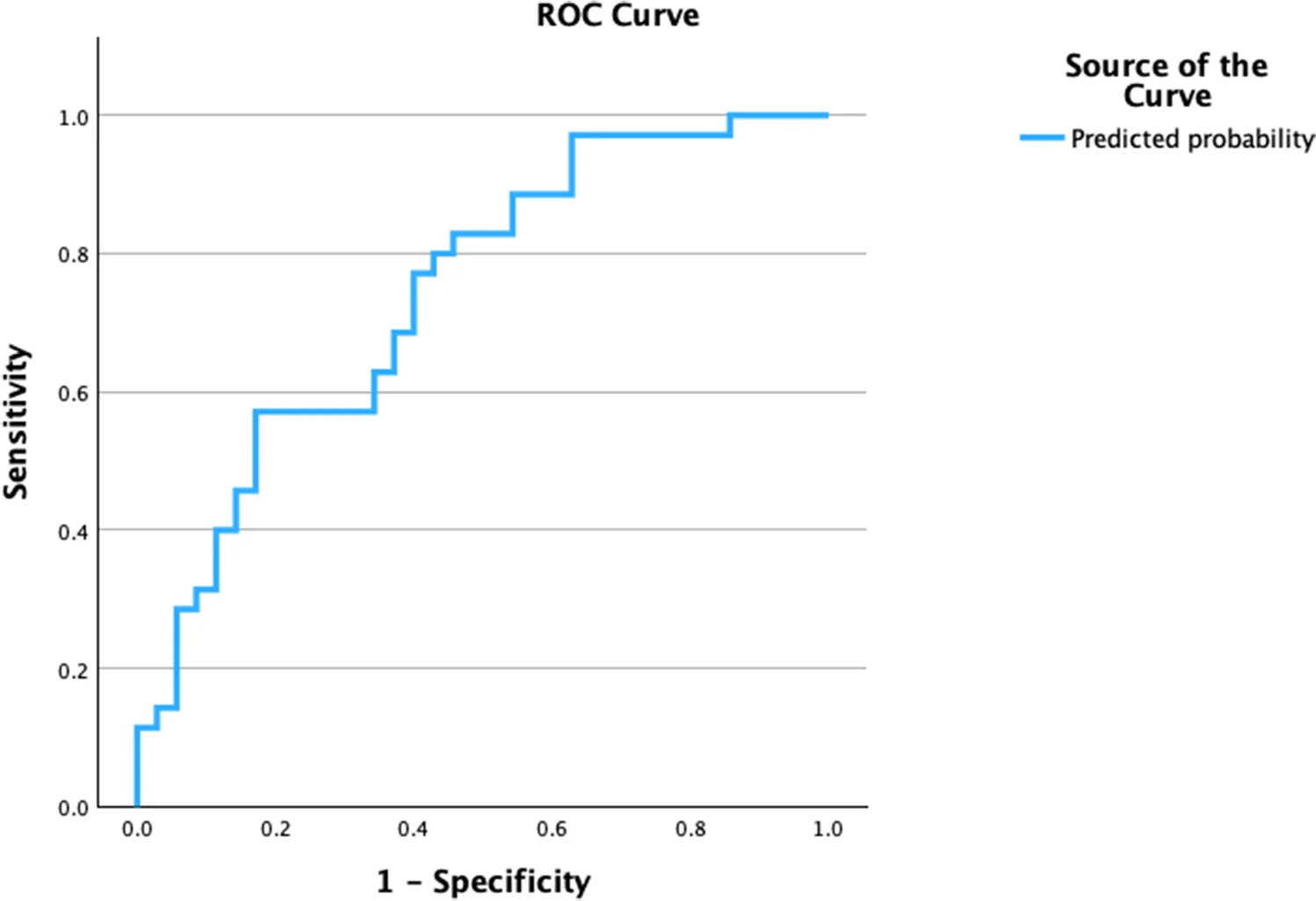
Receiver operating characteristic (ROC) curve of the logistic regression model predicting right-sided cervical rotational malposition based on mean left- and right-sided lateral atlantodental distance measurements (AUC=0.74)

ROC analysis based on the predicted probabilities generated by this model yielded an AUC of 0.74 (95% CI (0.63–0.86), *P*<0.001), indicating moderate discriminatory performance (Fig. 2).

## Discussion

Radiographic imaging in early infancy is technically challenging due to anatomical and behavioral factors of infants [29]. Despite these constraints, the present study demonstrated excellent inter-rater reliability for both right- and left-sided lateral atlantodental distance measurements, supporting the technical reproducibility and objectivity of the method, including when applied by non-radiologist raters. The low SEM further supports high measurement precision with minimal impact of random error [25, 29].

In infants with left-sided malposition, lateral atlantodental distance was significantly larger on the left side; in infants, with right-sided malposition, lateral atlantodental distance was larger on the right side. These side-specific differences were statistically significant but showed small to moderate effect sizes, indicating a measurable yet limited anatomical reflection of positional asymmetry. The asymmetry variable (AsymmetryOverall) showed both positive and negative values within clinically defined subgroups. This inconsistency may be explained by the fact that many side-to-side differences were close to the measurement error (SEM 0.62 mm; MDC 1.73 mm). Differences below this threshold cannot be reliably distinguished from random error and may therefore vary in direction despite an overall tendency greater lateral atlantodental distance values on the clinically affected side.

The findings of this study are in line with previous research showing that asymmetry of the lateral atlantodental interval can occur in pediatric populations and may represent normal anatomical or positional variation [20, 30–34]. Previous studies have reported lateral atlantodental interval asymmetry in asymptomatic children and emphasized that such findings should be interpreted with caution, particularly because head rotation and positioning can influence the relative relationship between the atlas and axis. This biomechanical and technical variability may partly explain why small side-to-side differences in lateral atlantodental distance frequently remained within the range of measurement error in the present study. Even if lateral atlantodental distance measurements themselves are technically reliable, variability introduced during image acquisition may limit the clinical relevance of small asymmetry values.

Taken together, these findings suggest that lateral atlantodental distance measurements should be interpreted as descriptive radiographic findings that may complement clinical assessment rather than as independent diagnostic markers of upper cervical spine dysfunction. Therefore, lateral atlantodental distance measurements should not be interpreted in isolation but considered alongside supplementary clinical indicators, including parental reports and findings from physical examination, to support informed clinical decision-making [35].

These considerations highlight the importance of integrating clinical and observational findings when evaluating cervical spine dysfunction. Radiological imaging of the cervical spine in infants should be approached with caution due to radiation exposure and used primarily to exclude structural pathologies when clinically indicated [36]. In accordance with the ALARA (As Low As Reasonably Achievable) principle, imaging should be reserved for well-justified cases following careful individual assessment of risks and benefits [37]. Given the complexity of cervical spine dysfunction in early infancy, a structured and interdisciplinary diagnostic approach remains essential. Clinical expertise from pediatricians, neurologists, and musculoskeletal specialists may support accurate evaluation and help avoid unnecessary imaging or overtreatment.

In addition, the study population consisted exclusively of infants referred for musculoskeletal evaluation and chiropractic care, which may limit the generalizability of the findings to broader infant populations or other clinical settings.

Despite the high inter-rater reliability and consistent measurement precision observed in this study, indicating that lateral atlantodental distance assessment is technically robust and reproducible even in an infant cohort, its diagnostic relevance in identifying upper cervical spine dysfunction remains uncertain due to the absence of validated clinical reference standards. Future research should investigate whether larger lateral atlantodental distance asymmetries exceeding the MDC threshold demonstrate greater clinical relevance or improved diagnostic discrimination. Further studies are warranted to better define the role of radiographic measurements as supportive findings within a broader clinical assessment of upper cervical spine dysfunction in infancy.

## Conclusions

Lateral atlantodental distance measurements demonstrated excellent inter-rater reliability, indicating that consistent measurements can be obtained from radiographic images of the upper cervical spine in infants. However, the relatively large minimal detectable change suggests that small side-to-side asymmetries cannot be reliably distinguished.

Rather than serving as a diagnostic or decision-making tool on its own, lateral atlantodental distance asymmetry appears to reflect a reproducible anatomical pattern that is associated with the clinically observed direction of cervical rotational malposition. This interpretation is consistent with the known biomechanical variability of the upper cervical spine. As such, lateral atlantodental distance measurements should be interpreted as descriptive radiographic findings that may complement clinical assessment, rather than as independent diagnostic markers or pathognomonic signs of upper cervical spine dysfunction.

## Supplementary Information

Below is the link to the electronic supplementary material.

ESM 1 (DOCX 78.1 KB)

ESM 2 (DOCX 1.07 MB)

## Data Availability

The datasets used and/or analysed during the current study are available from the corresponding author on reasonable request
