# Supplementary material for: Correlation of examiner judgement and radiological digital pictures in infants with upper cervical spine dysfunction: a cross-sectional study
Source: Pediatr Radiol. 2026 Jul 11;56(11):2573–80. doi: 10.1007/s00247-026-06718-8 (PMC13668896; doi:10.1007/s00247-026-06718-8)
Supplement: Supplementary file 1 [file 247_2026_6718_MOESM1_ESM.docx]

**Supplementary Material 1.** STROBE Statement—checklist of items that should be included in reports of observational studies

|  | Item No. | Recommendation | Page  No. | Relevant text from manuscript |
| --- | --- | --- | --- | --- |
| **Title and abstract** | 1 | (*a*) Indicate the study’s design with a commonly used term in the title or the abstract | Title page | Correlation of examiner judgement and radiological digital pictures in infants with upper cervical spine dysfunction: A cross-sectional study |
|  |  | (*b*) Provide in the abstract an informative and balanced summary of what was done and what was found | Abstract, Page 2 |  |
| Introduction | | | |  |
| Background/rationale | 2 | Explain the scientific background and rationale for the investigation being reported | Pages 4-5 |  |
| Objectives | 3 | State specific objectives, including any prespecified hypotheses | Page 5 | The aim of this cross-sectional study is to assess the inter-rater reliability (ICC) of the odontoid view in lateral lateral atlantodental distance measurements and to explore associations between lateral lateral atlantodental distance measurements and clinical presentation of infants with suspected upper cervical dysfunction in order to support clinical decision-making in early infancy. |
| Methods | | | |  |
| Study design | 4 | Present key elements of study design early in the paper | Page 5 |  |
| Setting | 5 | Describe the setting, locations, and relevant dates, including periods of recruitment, exposure, follow-up, and data collection | Pages 5 | The study followed a cross-sectional design. Right and left lateral lateral atlantodental distance measurements were obtained from radiographic images. Inter-rater agreement among three raters was analyzed, as well as the association between lateral lateral atlantodental distance measurements and the clinician’s assessment of cervical rotational malposition. Ethical approval was obtained from the local ethics committee (approval number blinded for review). The primary study was registered at a public trial registry (registration number blinded for review). Data collection took place between August 2021 and December 2023. |
| Participants | 6 | (*a*) *Cohort study*—Give the eligibility criteria, and the sources and methods of selection of participants. Describe methods of follow-up  *Case-control study*—Give the eligibility criteria, and the sources and methods of case ascertainment and control selection. Give the rationale for the choice of cases and controls  *Cross-sectional study*—Give the eligibility criteria, and the sources and methods of selection of participants | Page 6 | Radiological digital pictures of the odontoid of infants were assessed which were included in another study (blinded for review) fulfilling the following inclusion criteria: diagnosis of upper cervical spine dysfunction, age between three and twelve months and signed consent of the legal representative. Excluded were infants with known illness other than torticollis including neurological impairments, previous neck trauma, previous neck surgery and developmental delay. Radiological imaging was performed as part of the clinical routine before mobilization of the upper cervical spine. This was done in order to rule out contraindications for chiropractic treatment such as malformations, instabilities or severe pathologies of the infants’ upper cervical spine. During radiographic imaging, infants were gently supported by a parent, who stabilized the infant’s trunk and pelvis, to minimize movement while avoiding any direct fixation of the neck or shoulders. An x-ray technician gently held the infants` head within the x-ray window without overcorrecting the infants` head position. Afterwards the infants were animated to open the mouth by the present parent and/or the x-ray technician.  All included infants were referred to chiropractic care by their pediatrician, with a presumptive diagnosis of an upper cervical spine dysfunction. Any other underlying diagnoses such as neurological causes were ruled out before referral to chiropractic care by the pediatrician. No additional diagnostic procedures were executed during chiropractic care.  For the present study, infant age, sex, birth weight, birth length, the clinically assessed predominant side of cervical rotational malposition, and side-specific lateral atlantodental distance measurements were included in the analysis. Additional demographic, perinatal, and parental observational variables were collected as part of the primary study and are reported elsewhere (blinded for review) |
|  |  | (*b*) *Cohort study*—For matched studies, give matching criteria and number of exposed and unexposed  *Case-control study*—For matched studies, give matching criteria and the number of controls per case |  |  |
| Variables | 7 | Clearly define all outcomes, exposures, predictors, potential confounders, and effect modifiers. Give diagnostic criteria, if applicable | Page 7 | Radiograph adequacy and assessability were determined independently by each rater prior to lateral lateral atlantodental distance measurement, according to a predefined standard operating procedure (SOP) (see appendix 1). Raters were blinded to the ratings of each other. Rater one (18 years of clinical experience) and rater three (7 years of clinical experience) were clinicians specialized in musculoskeletal care. Rater two was a radiologist with 4 years of clinical expertise in specialized musculoskeletal imaging. Before image assessment, all raters completed two standardized training sessions focusing on the evaluation of upper cervical spine radiological digital images and on performing lateral lateral atlantodental distance measurements. Measurements were performed in accordance with the SOP to ensure standardized and reproducible assessment of the radiological images. Specifically, the outline of the odontoid process and the lateral masses of the atlas (C1) had to be clearly visible. The shortest distance between the lateral aspect of the dens (C2) and the medial border of the lateral mass of the atlas (C1) was measured on an anterioposterior radiograph (Fig. 1) (21). Distances between odontoid process and lateral masses of the atlas were recorded in millimeters for right lateral atlantodental distance and left lateral atlantodental distance. Measurements were performed using the MERLIN Diagnostic Workcenter (Phönix-PACS GmbH, Freiburg, Germany). |
| Data sources/ measurement | 8* | For each variable of interest, give sources of data and details of methods of assessment (measurement). Describe comparability of assessment methods if there is more than one group | Page 7 | See above |
| Bias | 9 | Describe any efforts to address potential sources of bias |  | N/A |
| Study size | 10 | Explain how the study size was arrived at | Page 5 | Radiological digital pictures of the odontoid of infants were assessed which were included in another study (blinded for review) fulfilling the following inclusion criteria: diagnosis of upper cervical spine dysfunction, age between three and twelve months and signed consent of the legal representative. Excluded were infants with known illness other than torticollis including neurological impairments, previous neck trauma, previous neck surgery and developmental delay. Radiological imaging was performed as part of the clinical routine before mobilization of the upper cervical spine. This was done in order to rule out contraindications for chiropractic treatment such as malformations, instabilities or severe pathologies of the infants’ upper cervical spine. During radiographic imaging, infants were gently supported by a parent, who stabilized the infant’s trunk and pelvis, to minimize movement while avoiding any direct fixation of the neck or shoulders. An x-ray technician gently held the infants` head within the x-ray window without overcorrecting the infants` head position. Afterwards the infants were animated to open the mouth by the present parent and/or the x-ray technician.  All included infants were referred to chiropractic care by their pediatrician, with a presumptive diagnosis of an upper cervical spine dysfunction. Any other underlying diagnoses such as neurological causes were ruled out before referral to chiropractic care by the pediatrician. No additional diagnostic procedures were executed during chiropractic care. |

| Quantitative variables | 11 | Explain how quantitative variables were handled in the analyses. If applicable, describe which groupings were chosen and why | Pages 8 | Study data was managed using REDCap Electronic Data Capture systems (Vanderbilt University, TN, USA) [21, 22]. Statistical analysis was performed using SPSS (Version 30, IBM, Armonk, NY USA). Demographics were determined and descriptive statistics of the sample were calculated. Paired-sampled t-tests were conducted to assess differences in lateral lateral atlantodental distance between right and left sides [23]. In all t-tests, values in parentheses denote degrees of freedom. In addition, the data were subdivided based on the side of the infant’s cervical malposition (right- or left-sided), allowing side-specific comparisons. Inter-rater reliability (agreement between raters) was assessed using intraclass correlation coefficient (ICC) based on mean-rating (k=3), absolute-agreement, two-way mixed-effects model. The ICC was calculated separately for the measurements of the LADD on the right and left sides. Values greater than 0.9 represent excellent reliability, 0.75-0.9 good agreement, 0.5-0.75 moderate agreement and under 0.5 poor agreement [24]. Furthermore, standard error of measurement (SEM) (SEM = SD √1 − ICC) was calculated to quantify the expected variability in individual scores due to measurement error [25]. Minimal detectable change (MDC) (MDD = 1.96 ∗ √2 ∗ SE) were calculated to assess absolute reliability. Significance level was set at α = 0.05 [26, 27]. In a separate analysis, the association between lateral atlantodental distance and cervical rotational malposition was examined. The data were stratified based on the clinically affected side. Specifically, left and right lateral atlantodental distance values were compared in infants with left-sided malposition and with right-sided malposition separately. This allowed for a side-specific analysis of asymmetry in relation to clinical findings.  For inferential analysis, logistic regression and receiver operating characteristic (ROC) analyses were performed to explore whether lateral atlantodental distance measurements were associated with the clinically assessed side of cervical rotational malposition [27, 28]. Right-sided cervical rotational malposition was specified as the outcome of interest in the logistic regression and ROC analyses. Mean lateral atlantodental distance values were calculated using all available measurements from the three raters. Cases with missing measurements from one or two raters were retained for analyses of mean left and right lateral atlantodental distance values using the available measurements. ICC analyses were restricted to complete cases with measurements available from all three raters.  To quantify side-specific asymmetry, the difference between the affected and unaffected side was calculated (AsymmetryOverall). Positive values indicated greater lateral atlantodental distance on the clinically affected side, whereas negative values indicated greater lateral atlantodental distance on the clinically unaffected side. |
| --- | --- | --- | --- | --- |
| Statistical methods | 12 | (*a*) Describe all statistical methods, including those used to control for confounding | Pages 8 | See above |
|  |  | (*b*) Describe any methods used to examine subgroups and interactions | Pages 8 | See above |
|  |  | (*c*) Explain how missing data were addressed | Pages 8 | See above |
|  |  | (*d*) *Cohort study*—If applicable, explain how loss to follow-up was addressed  *Case-control study*—If applicable, explain how matching of cases and controls was addressed  *Cross-sectional study*—If applicable, describe analytical methods taking account of sampling strategy |  | Not applicable |
|  |  | (*e*) Describe any sensitivity analyses |  |  |
| Results | | | | |
| Participants | 13* | (a) Report numbers of individuals at each stage of study—eg numbers potentially eligible, examined for eligibility, confirmed eligible, included in the study, completing follow-up, and analysed | N/A |  |
|  |  | (b) Give reasons for non-participation at each stage | N/A |  |
|  |  | (c) Consider use of a flow diagram | N/A |  |
| Descriptive data | 14* | (a) Give characteristics of study participants (eg demographic, clinical, social) and information on exposures and potential confounders | Page 10 | Seventy-two infants were included (mean age 145.4 (SD 29) days, mean birth weight 3303.6 (SD 542.3) g, mean birth length 49.3 (SD 2.8) cm). Forty-seven infants (65%) were male and twenty-five (35%) female.  The clinician rated thirty-five (49%) infants as having a rotational malposition of the head to the right side and thirty-seven (51%) to the left side. |
|  |  | (b) Indicate number of participants with missing data for each variable of interest | Page 10 | Due to insufficient radiographic image quality, lateral atlantodental distance measurements could not be obtained in seven cases by rater 1, eleven cases by rater 2, and three cases by rater 3. Two radiographs were excluded entirely because image quality was insufficient for lateral atlantodental distance assessment by any rater. Both excluded cases had been clinically classified as left-sided cervical rotational malposition. Consequently, the final study sample for the side-specific lateral atlantodental distance analysis included 70 infants, comprising 35 with left-sided and 35 with right-sided cervical rotational malposition. |
|  |  | (c) *Cohort study*—Summarise follow-up time (eg, average and total amount) |  |  |
| Outcome data | 15* | *Cohort study*—Report numbers of outcome events or summary measures over time |  |  |
|  |  | *Case-control study—*Report numbers in each exposure category, or summary measures of exposure |  |  |
|  |  | *Cross-sectional study—*Report numbers of outcome events or summary measures | Page 10 ff |  |
| Main results | 16 | (*a*) Give unadjusted estimates and, if applicable, confounder-adjusted estimates and their precision (eg, 95% confidence interval). Make clear which confounders were adjusted for and why they were included | Page 10 ff | In infants with a left-sided rotational malposition (*n* = 35), the left lateral atlantodental distance was significantly larger than the right. A paired-samples t-test revealed a significant side difference, t(34) = 3.11, *P* = 0.004, with a mean difference of 1.11 mm (95% CI: 0.39 to 1.84 mm) and a moderate effect size (Cohen’s d = 0.53), supporting the presence of side-specific asymmetry within this subgroup. In infants with a right-sided rotational malposition (*n* = 35), the right lateral atlantodental distance was significantly greater than the left. A paired-samples t-test revealed a significant side difference, t(34) = -2.08, *P*=0.05, with a mean difference of -0.62 mm (95% CI:-1.22 to -0.01 mm) and a small to moderate effect size (Cohen’s d=0.35), indicating side-specific asymmetry within this subgroup. Descriptive statistics for the lateral atlantodental distance measurements are presented in Table 1. The values illustrate the observed side-specific differences in lateral atlantodental distance according to the side of cervical rotational malposition. In infants with a left-sided malposition, the lateral atlantodental distance was greater on the left side, whereas in infants with a right-sided malposition, the lateral atlantodental distance was greater on the right side. The asymmetry variable (AsymmetryOverall), calculated as the difference between the affected and unaffected side, showed both positive and negative values within both clinically defined subgroups. **3.3 Interrater reliability, standard error of measurement and minimal detectable change** Based on 60 complete cases, interrater reliability (ICC) was excellent for both left-sided (0.91 [95 % CI: 0.86 – 0.95]) and right-sided (0.91 [95 % CI: 0.86 – 0.94]) lateral atlantodental distance measurements. For the left-side, SEM was 0.62 mm, and the minimal detectable change (MDC) was 1.73 mm. For the right-side, SEM was also 0.62 mm and MDC was 1.72 mm. These thresholds represent the minimum difference required to be confident that an observed change exceeds measurement error and reflects a true difference. **3.4 Binary logistic regression** Binary logistic regression was performed in 70 infants with available side-specific mean lateral atlantodental distance measurements. The analysis demonstrated an association between side-specific LADD measurements and clinically assessed cervical rotational malposition. Only the mean right-sided lateral atlantodental distance was a significant predictor of clinically assessed right sided malposition (OR 1.80, 95% CI 1.14–2.84, *P* = 0.01), whereas the mean left-sided lateral atlantodental distance was not statistically significant (Table 2). **Table 2** Binary logistic regression model predicting right-sided cervical rotational malposition **Predictor** \| **OR** \| **95% CI** \| ***p*-value**; **Mean left lateral atlantodental distance** \| 0.70 \| 0.45-1.09 \| 0.12; **Mean right lateral atlantodental distance** \| **1.80** \| **1.14-2.84** \| **0.01** *OR,* odds ratio; *CI*, confidence interval; Significant results are highlighted in bold ROC analysis based on the predicted probabilities generated by this model yielded an AUC of 0.74 (95% CI (0.63 – 0.86), *P* < 0.001), indicating moderate discriminatory performance (Fig. 2). |
|  |  | (*b*) Report category boundaries when continuous variables were categorized | N/A |  |
|  |  | (*c*) If relevant, consider translating estimates of relative risk into absolute risk for a meaningful time period | N/A |  |

Continued on next page

| Other analyses | 17 | Report other analyses done—eg analyses of subgroups and interactions, and sensitivity analyses | Page 11 ff | See above |
| --- | --- | --- | --- | --- |
| Discussion | | | | |
| Key results | 18 | Summarise key results with reference to study objectives | Page 13 | In infants with left-sided malposition lateral atlantodental distance was significantly larger on the left side; in infants, with right-sided malposition, lateral atlantodental distance was larger on the right side. These side-specific differences were statistically significant but showed small to moderate effect sizes, indicating a measurable yet limited anatomical reflection of positional asymmetry. The asymmetry variable (AsymmetryOverall) showed inconsistent results with positive and negative values within subgroups defined by right- or left-sided rotational malposition. This inconsistency might be explained by the fact that the average side-to-side differences were often close to the measurement error (SEM 0.62 mm; MDC 1.73 mm). Differences below this threshold cannot be reliably distinguished from random error, which likely caused the calculated asymmetry index to cross zero in both directions. Consequently, no consistent directional pattern emerged, and both logistic regression and ROC analyses showed poor discriminatory performance without identifying any clinically useful threshold.  The overall findings of this study therefore suggest that, although lateral atlantodental distance measurements are technically reliable, lateral atlantodental distance asymmetry alone does not provide a sufficiently robust basis to determine the side of cervical dysfunction in infants. Therefore, the interpretation of lateral atlantodental distance measurements requires careful consideration of both technical reliability and clinical relevance. |
| Limitations | 19 | Discuss limitations of the study, taking into account sources of potential bias or imprecision. Discuss both direction and magnitude of any potential bias | Page 13ff | The findings of this study are in line with previous research showing that asymmetry of the lateral atlantodental interval can occur in pediatric populations and may represent normal anatomical or positional variation [20, 30–34]. Previous studies have reported lateral atlantodental interval asymmetry in asymptomatic children and emphasized that such findings should be interpreted with caution, particularly because head rotation and positioning can influence the relative relationship between the atlas and axis. This biomechanical and technical variability may partly explain why small side-to-side differences in lateral atlantodental distance frequently remained within the range of measurement error in the present study. Even if lateral atlantodental distance measurements themselves are technically reliable, variability introduced during image acquisition may limit the clinical relevance of small asymmetry values.  Taken together, these findings suggest that lateral atlantodental distance measurements should be interpreted as descriptive radiographic findings that may complement clinical assessment rather than as independent diagnostic markers of upper cervical spine dysfunction. Therefore, lateral atlantodental distance measurements should not be interpreted in isolation but considered alongside supplementary clinical indicators, including parental reports and findings from physical examination, to support informed clinical decision-making [35].  These considerations highlight the importance of integrating clinical and observational findings when evaluating cervical spine dysfunction. Radiological imaging of the cervical spine in infants should be approached with caution due to radiation exposure and used primarily to exclude structural pathologies when clinically indicated [36]. In accordance with the ALARA (As Low As Reasonably Achievable) principle, imaging should be reserved for well-justified cases following careful individual assessment of risks and benefits [37]. Given the complexity of cervical spine dysfunction in early infancy, a structured and interdisciplinary diagnostic approach remains essential. Clinical expertise from pediatricians, neurologists, and musculoskeletal specialists may support accurate evaluation and help avoid unnecessary imaging or overtreatment.  In addition, the study population consisted exclusively of infants referred for musculoskeletal evaluation and chiropractic care, which may limit the generalizability of the findings to broader infant populations or other clinical settings.  Despite the high inter-rater reliability and consistent measurement precision observed in this study, indicating that lateral atlantodental distance assessment is technically robust and reproducible even in an infant cohort, its diagnostic relevance in identifying upper cervical spine dysfunction remains uncertain due to the absence of validated clinical reference standards. Future research should investigate whether larger LADD asymmetries exceeding the MDC threshold demonstrate greater clinical relevance or improved diagnostic discrimination. Further studies are warranted to better define the role of radiographic measurements as supportive findings within a broader clinical assessment of upper cervical spine dysfunction in infancy. |
| Interpretation | 20 | Give a cautious overall interpretation of results considering objectives, limitations, multiplicity of analyses, results from similar studies, and other relevant evidence | Page 13ff | See above |
| Generalisability | 21 | Discuss the generalisability (external validity) of the study results | Page 14 | In addition, the study population consisted exclusively of infants referred for musculoskeletal evaluation and chiropractic care, which may limit the generalizability of the findings to broader infant populations or other clinical settings.  Despite the high inter-rater reliability and consistent measurement precision observed in this study, indicating that lateral atlantodental distance assessment is technically robust and reproducible even in an infant cohort, its diagnostic relevance in identifying upper cervical spine dysfunction remains uncertain due to the absence of validated clinical reference standards. Future research should investigate whether larger lateral atlantodental distance asymmetries exceeding the MDC threshold demonstrate greater clinical relevance or improved diagnostic discrimination. Further studies are warranted to better define the role of radiographic measurements as supportive findings within a broader clinical assessment of upper cervical spine dysfunction in infancy. |
| Other information | |  | | |
| Funding | 22 | Give the source of funding and the role of the funders for the present study and, if applicable, for the original study on which the present article is based | Page 18 | Not applicable. |

*Give information separately for cases and controls in case-control studies and, if applicable, for exposed and unexposed groups in cohort and cross-sectional studies.

**Note:** An Explanation and Elaboration article discusses each checklist item and gives methodological background and published examples of transparent reporting. The STROBE checklist is best used in conjunction with this article (freely available on the Web sites of PLoS Medicine at http://www.plosmedicine.org/, Annals of Internal Medicine at http://www.annals.org/, and Epidemiology at http://www.epidem.com/). Information on the STROBE Initiative is available at www.strobe-statement.org.
