# Supplementary material for: Correlation of examiner judgement and radiological digital pictures in infants with upper cervical spine dysfunction: a cross-sectional study
Source: Pediatr Radiol. 2026 Jul 11;56(11):2573–80. doi: 10.1007/s00247-026-06718-8 (PMC13668896; doi:10.1007/s00247-026-06718-8)
Supplement: Supplementary file 2 [file 247_2026_6718_MOESM2_ESM.docx]

**Standard operating procedure for measuring the lateral atlantodental distances**

**Asses the quality of the open-mouth view**

Verify that the outline of the odontoid process and of the lateral masses is clearly visible. Ensure high-quality imaging with correct patient positioning. Alignment is not an exclusion criteria as long the dens and the massae lateralis is visible.

**Measuring the distance between the odontoid process and the lateral masses of C1**

Measure the points of the shortest distance between the most medial part of the lateral mass to the dens with the distance tool in Merlin^1^. Identify the visible vertical line of the outside of the dens. Select the shortest distance between: The most medial part of the lateral mass of C1 and the point where the borders of the odontoid process are most parallel to the axis of the odontoid process (dens). The measurement should be taken strictly horizontally as seen in the image, rather than in relation to the patient’s anatomical orientation. Ensure the measurement is taken in the **middle part** of the dens, not at the superior or inferior portions. Refer to Figure 2 (white arrows) or Figure 3 (line indication) as a guide.

R :

L :

**R**

**L**

***Figure 1*** Measurement of lateral atlantodental distance on anteroposterior radiograph of an infant. The image is depicted in radiological convention – anatomical right appears on the left and anatomical left on the right. Lateral atlantodental distance was measured between the lateral border of the odontoid process and the medial border of the lateral mass of the atlas (C1), using radiographic data obtained in this study and processed with digital software (MERLIN Diagnostic Workcenter, Phönix-PACS GmbH, Freiburg, Germany). Anatomical sides are indicated by the markers “R” and “L”

**RedCap** The distances on either side of the odontoid process are to be measured and recorded in mm as two separate datapoints right lateral atlantodental distance and left lateral atlantodental distance.

The white arrows (Figure 2) or line (Figure 3) in the picture below also show at which point the distance should be measured:

**Figure 3** Zhang XB, Luo C, Li M, Zhang X, Hui H, Zeng Q, Li TY, Zhang DW, Zhang YY, Wang C, Liu CK, Liu X, Qu XY, Cao YJ, Zhou H, Weng LQ. Clinical significance of imaging findings for atlantoaxial rotatory subluxation in children. Turk J Med Sci. 2018 Apr 30;48(2):332-338. doi: 10.3906/sag-1707-137. PMID: 29714448.

***Figure 2*** Adib O, Berthier E, Loisel D, Aubé C. Pediatric cervical spine in emergency: radiographic features of normal anatomy, variants and pitfalls.
Skeletal Radiol. 2016 Dec;45(12):1607-1617. doi: 10.1007/s00256-016-2481-9.
Epub

**Reference**

1.	Rojas CA, Hayes A, Bertozzi JC, Guidi C, Martinez CR. Evaluation of the C1-C2 articulation on MDCT in healthy children and young adults. AJR Am J Roentgenol. 2009 Nov;193(5):1388-92. doi: 10.2214/AJR.09.2688. PMID: 19843758.

2016 Sep 20. PMID: 27650073.
